# Randomised trials relevant to mental health conducted in low and middle-income countries: protocol for a survey of studies published in 1991, 1995 and 2000 and assessment of their relevance

**DOI:** 10.1186/1471-244X-6-40

**Published:** 2006-09-26

**Authors:** Rebecca J Syed Sheriff, Mahesh Jayaram, Prathap Tharyan, Lelia Duley, Clive E Adams

**Affiliations:** 1 Unidad Epidemiologia Clinica, Hospital San Ignacio, Santafe de Bogota, Colombia; 2 Cochrane Schizophrenia Group Academic Unit of Psychiatry and Behavioural Sciences, University of Leeds, UK; 3 South Asian Cochrane Network; Prof. Bhooshanam V Moses Centre for Clinical Trials and Evidence Based Medicine, Christian Medical College, Vellore, India; 4 Nuffield Department of Medicine, University of Oxford, UK

## Abstract

**Background:**

A substantial proportion of the psychiatric burden of disease falls on the world's poorest nations. Despite this, relatively little is known about the quality and content of clinical research undertaken in these countries, or the relevance of the interventions evaluated and specifically that of randomised trials.

This project aims to survey the content, quality and accessibility of a sample of trials relevant to mental health conducted within low and middle-income countries; to compare these with studies conducted in high-income countries; and to assess their relevance for the needs of low and middle-income countries.

**Methods:**

An extensive search for all trials, or possible trials, published in 1991, 1995 and 2000 with participants in low and middle-income countries has already been conducted. Studies evaluating prevention or treatment of a mental health problem within these three years will be identified and further searches conducted to assess completeness of the initial search. Data on study quality and characteristics will be extracted from each report. Accessibility will be estimated based on whether each citation is available on MEDLINE. Trials relevant to schizophrenia will be compared with a random sample of schizophrenia trials from high-income countries in the same years. Topics covered by the trials will be compared with the estimated burden of disease.

**Conclusion:**

Trials and systematic reviews of trials are the gold standard of evaluation of care and increasingly provide the basis for recommendations to clinicians, to providers of care and to policy makers. Results from this study will present the first assessment of the scope, quality and accessibility of mental health trials in low and middle-income countries.

## Background

Most of the global burden of mental illness falls to the poorest nations, where 80% of world's population live [1]. Major depression is now a leading cause of disability throughout the world and ranks fourth in the ten leading causes of the global burden of disease (measured using 'Disability Adjusted Life Years' – DALYS) [2,3].

According to the World Health Organisation (WHO) "Most low/middle income countries devote less than 1% of their health expenditure to mental health. Health policies, legislation, community care facilities and treatments for mentally ill people are therefore dismally short of resources" [4]. Most evaluative research is conducted in high-income countries, and then applied to low and middle-income countries [5].

The relevance of existing research to the world's poorer nations is questionable. Only 10% of the total spent on health research is directed towards the diseases which are responsible for 90% of the global burden of disease [6]. Health funding is generally provided by higher income countries whereas much of the global burden of disease is in the less rich nations. In fact, expenditure on health research in many low and middle-income countries is largely unaccounted for [7]. Interventions evaluated in trials conducted in high-income countries tend to be unaffordable or unavailable to those in poorer countries. For the world to derive maximum benefit from health research, the balance of research between rich and poor countries needs to shift radically [8].

In mental health, the number of trials conducted in any one country seems to be correlated with the wealth of that nation: Gross Domestic Product (GDP) has been shown to be more correlated with the number of schizophrenia trials than with population size, GDP per capita, or number of telephones/100 people [9]. If this correlation is extended to other areas of mental health, the number of mental health trials would be considerably lower in poor countries than in rich nations. Furthermore, there is evidence that lower income countries are under-represented in psychiatric literature. In one survey of high impact journals, 90% of the literature was derived from "Euro-American" societies [10], in addition a search of the ISI Web Science database showed that lower and middle-income countries contributed only 6% of the mental health literature [11]. It is subsequently likely that less mental health research activity takes place in poorer countries and, in addition, that reports of these trials are less accessible.

As part of a project to improve access to pragmatic trials in developing countries [12] randomised trials, or possibly randomised trials, with participants from low or middle-income countries published during 1991, 1995 or 2000 have been identified. This protocol is for a survey to describe in detail the sub-set of these trials relevant to mental health care and to describe their quality, content, accessibility and relevance.

The utility of trials is largely dependent upon their relevance, quality and accessibility. How many trials relevant to mental health that include participants from low and middle-income countries is unknown. Of the trials relevant to mental health conducted at least in part within low and middle income countries, little is known about their relevance to the health needs in those countries. The quality of these studies, their accessibility both locally and internationally, and how these vary according to income or geographical region are also unclear.

This study will survey reports of trials relevant to mental health with participants from low and middle-income countries published in 1991, 1995 and 2000 with the aim of describing their content, quality and trends over time and how these vary according to income and broad geographical region. Other objectives are:

• to compare the topics covered with those in the WHO estimates of burden of disease;

• to ascertain the availability of citations on Medline as an indicator of accessibility (Medline was chosen as it is widely used and available freely world wide); and

• for schizophrenia trials, to compare the content, quality and accessibility with a sample of those from high income countries published in the same years.

## Methods

The search for randomised trials, or possible randomised trials, relevant to health care, with participants from low and/or middle income countries and published in the years 1991, 1995 or 2000 was performed for the main Practihc project.

According to the World Bank [13] each country is classified as low-income, middle-income (subdivided into lower middle or upper middle), or high-income based on its Gross National Income (GNI) per capita. In low-income countries the GNI is $745 or less, in lower middle-income countries it is $746-$2,975, in upper middle-income countries it is $2, 976- 9, 205, and in high-income countries it is 9, 206 or more. Each country is reclassified on a yearly basis.

The sample years 1991, 1995 and 2000 were chosen in 2003. This was to include a 10-year spread at 5-year intervals. This time period ended 3 years prior to the search so that papers published in those years would have had the chance to reach the databases. This Practihc project has made available full text copies for over 90% of the citations published in these three years.

In brief, the methods for the Practihc search initially involved searching the Cochrane Library's CENTRAL database but expanded to other databases likely to include studies from low and middle-income countries when it became clear that CENTRAL was not a complete source of such studies. The strategy was as follows. Records from the Cochrane Library's CENTRAL database (Issue 3, 2004) published in 1991, 1995 and 2000 were downloaded into a bibliographic management package [14]. The ProCite database was then searched for names of low and middle-income countries and terms used to identify lower income countries, such as 'Africa'. The list of countries was obtained from the World Bank for each of the three years. Wherever possible, country names were searched for in relevant languages and using names relevant for that time period. Further searches used terms for conditions specific to low and middle-income countries, such as leprosy, leshmaniasis, malaria, filarial and tuberculosis. This helped to identify eligible citations not picked up in searches using country names. The search results were transferred into an MS Access database [15].

To assess the completeness of CENTRAL for trials conducted in low and middle income countries, additional searches of a range of bibliographic databases (see Additional File 1) were conducted. The search for databases was intended to be as comprehensive as possible. This involved utilising personal contacts, WHO web links as well as using the international search engine Google [16] for the name of the populous country, bibliographic and medical terms. The search strategies used within these databases depended on the constraints of the search interface of each particular database. The simple terms "randomised" and "double blind" were searched in all of the databases, additional terms were added if the database allowed. Potentially eligible trials found in these databases were checked to exclude duplicate citations. All records were printed out and manually checked for eligibility. This was done by LD, CEA and MJ. Each trial was checked individually and with a 10% overlap. Records that were clearly not a randomised trial according to the definitions in the Cochrane Handbook [17] were excluded, as were those that clearly did not have any participants from a low or middle-income country. For all remaining citations, full text copies were sought and converted, where necessary, to PDF files.

### Eligibility criteria

For this project, articles in the main Practihc sample will be considered eligible for the survey of mental health trials if they meet the following criteria:

• As for the main Practihc project, studies must be randomised trials or possible randomised trials published in 1991, 1995 or 2000 with participants from low or middle income countries as defined by the World Bank. According to the definitions in the Cochrane Handbook randomised trials, quasi randomised trials and controlled clinical trials which could possibly be randomised will be included. We will exclude studies with historical controls and case control studies.

• Participants in the trials must be people who either had a mental health problem according to the WHO compiled classification system (ICD-10) for psychiatric and behavioural disorders, or were people identified as being at increased risk of developing a mental health problem, which an intervention could potentially prevent.

### Search strategy within the Practihc database

The main Practihc sample will be searched using the title, keyword and abstract within the MS Access database. Word roots based on Mesh headings within "Mental Health" will be utilised with extra terms in Spanish, Portuguese and French (see Additional File 2). This is intended to be comprehensive. Terms will be added from the ICD-10 and will be used if the yield increases.

All authors of citations identified in this search will be sought for within MEDLINE, to identify further trials. This strategy is based on the assumption that the same people may have been authors for other eligible trials. The purpose is to assess the completeness of the original search and identify any reports missed. This is thought to be a more useful strategy than checking reference lists of eligible articles which are less likely to have come from low or middle income countries and are less likely to have come from the sample year.

To assess whether the search for mental health studies failed to identify eligible studies (false negatives), all citations with reports in English will be manually inspected for eligibility.

In addition, all of the reports in Russian, Ukrainian, Romanian and Spanish will be manually checked for eligibility. Reports in Chinese, Portuguese and Polish for which data has been extracted for the Practihc project will be checked for potentially eligible studies using the extracted data under the items "problems being addressed" and "outcomes" (see Additional File 4).

### Assessment of potentially eligible studies

Studies identified in the search strategy will be manually inspected for eligibility for entry into this survey. This inspection will be done by two investigators working independently in accordance with the criteria stated earlier. Discrepancies will be resolved by discussion. Any remaining disagreements will be discussed with a third author and any remaining uncertainty will lead to the inclusion of a study.

For trials with only the abstract or title in English, resolving discrepancies may require translation of the full text and this will be carried out if possible. Where not possible, the trial will be included for further assessment by the associate researcher at the point of data extraction.

### Selection of the trials of people with schizophrenia

These will be identified using information extracted about the problem being addressed within each study and the outcomes reported (see below). Eligible trials will be those in which participants either had schizophrenia according to (ICD-10), or were people identified as being at increased risk of developing schizophrenia which an intervention could potentially prevent.

The register of trials maintained by the Cochrane Schizophrenia Group will also be used to identify trials relevant to people with schizophrenia conducted in high-income countries in the same years. This register is intended to be as comprehensive as possible and therefore should be representative.

A sub-sample of the same number of trials from high income countries as those identified for low and middle income countries will be randomly selected from the Cochrane Schizophrenia Group Register for comparison of additional variables related to quality and content.

### Data extraction

For studies in English, data is being extracted for the main Practihc project by researchers at the Christian Medical College, Vellore, India who have been trained by and are under the supervision of PT. Site visits have been conducted by LD to ensure consistency of data extraction, and assist with quality control. Data extraction for selected other languages is being conducted in Argentina and the UK. LD has trained those extracting in Russian, Chinese and Ukrainian. There is also a manual of procedures for data extraction and entry used at all sites. Data being extracted includes information about the authors, methods, participants, interventions, outcomes, and also funding sources and ethics approval. Measures related to quality include randomisation, sequence generation, allocation concealment, blinding of the intervention and completeness of follow up. Where possible this data is extracted from the full report. If this is unavailable the abstract is used. Data extraction forms were developed following piloting within the Practihc group and the Cochrane Schizophrenia Group (see Additional File 3). These definitions were operationalised by how the trial was reported e.g. classification as single or double blind depends upon how it is explicitly reported in the paper, rather than on assumptions or extrapolations made by the data extractor. Problems being addressed were therefore written as they had been in the report, these can then be coded into MeSH terms at the data entry stage. However, some items that were not explicitly mentioned in the article such as site could be coded as "hospital" if this must obviously have been the case eg in a trial of ECT. This data is being entered into Meerkat.

For the purposes of this study, extra information will also be extracted from all of the mental health articles identified (see Additional File 4). This extra information will be:

• age groups according to the WHO classification;

• classification of validity according to concealment of allocation, graded as adequate concealment, uncertain and clearly inadequate as described by Juni 2001 [18] and also, if a prevention trial

• the type of prevention; primary prevention or relapse prevention

This information will be extracted by RJSS for studies in English and Spanish and by associate researchers for reports in other languages with training and supervision from RJSS. All associate researchers use a manual of procedures for data extraction.

Data extracted from the trials identified in this study will be compared with the data that has already been extracted for the main Practihc project. At least 10% of reports will be extracted in duplicate for the original Practihc data. If this has not ready taken place for the Practihc study this will be undertaken within this mental health project. In addition, 10% of the extra mental health data will be double data extracted by MJ for reports in English or by associate researchers for reports in other languages. The degree of concordance for each of the variables will be calculated according to predetermined criterion. For each variable a percentage agreement of above 80% will be accepted. If the percentage agreement for any variable is below 80%, possible reasons will be investigated and further data extracted to ensure quality control. All data will be entered into a form within Meerkat so that it can be compared with the data extracted into Meerkat for the main Practihc study.

Data will also be extracted from the comparison sample of schizophrenia trials from high-income countries by MJ (see Additional File 5) with an additional 10% extracted by RJSS working independently. The degree of concordance for each of the variables will be calculated. Again, a percentage agreement of above 80%, according to predetermined criterion, will be accepted. The same protocol as explained above will be followed if the percentage agreement is below this. The accessibility of each report will be estimated by ascertaining whether it is indexed in Pub med.

### Analyses

The following analyses will be conducted (for detailed dummy tables see Additional File 6):

Studies will be described in terms of type of report, country of first author, country/ies of recruitment, language of full report, age of participants, sex of participants, problems being addressed, main aims, number randomised, type of intervention, setting of interventions, site of intervention, duration of intervention, follow up duration, funding source, funding country, ethics approval, consent, concealment of allocation, sequence generation, blinding, number of participants with outcome data and types of outcome and accessibility (tables 1–24).

Trends over the 10-year time frame will be described for numbers of citations and total numbers of participants randomised in trials. This will be done for all low and middle income-countries and according to income of country of recruitment, using the World Bank definition (low, lower middle, upper middle and high), and broad geographic region according to the WHO definition (e.g. Latin America and Caribbean), (figures 1, 2, 3, 4, 5, 6).

Trends over time will also be described for grading of concealment of allocation, types of intervention biological or psycho/social, and accessibility (figures 7, 8, 9, 10).

Comparison of income groups and geographic regions of countries of recruitment will occur for grading of concealment of allocation, accessibility and number randomised (see dummy tables 25–27).

The association between the following variables will also be assessed (see dummy tables 28–30):

• accessibility with language of publication and quality;

• quality as indicated by grading of concealment of allocation with accessibility; and

• source of funding with concealment of allocation and language.

### Comparison with studies from high-income countries

The sub sample of studies for trials in schizophrenia will be compared with the comparison sample of studies from high-income countries. Comparison of allocation concealment grading, size of study according to number randomised, type of intervention and accessibility will be carried out (tables 31–34). A simple statistical analysis will be carried out in order to compare differences in means and proportions of these variables.

### Comparison with the global burden of disease

Global burden of disease estimates are available from the WHO for 1990 and 2000–2002. According to advice from the WHO, differences in the estimates between these years are mainly due to improvements in information gathering rather than actual changes in need. The most recent information is the most accurate. Global burden of disease estimates for mortality and YLD (years lost to disability) are available for both for economic groups (high, upper middle, lower middle and low) and broad geographic regions e.g. Latin America and Caribbean, excluding high-income countries (see Additional File 7). These estimates are further categorised by cause e.g. unipolar depression.

A "research: need" ratio for all mental health related causes will be calculated. This will be done by dividing the number of people randomised in trials by the YLD. The research: need ratio will be calculated for economic groups and geographic regions and also for each individual cause, each age group and each gender group (see dummy table 37). The findings will be compared with each other in order to investigate which disorders and populations are the focus of a relatively high or low research activity.

## Discussion

Trials and systematic reviews of trials are the gold standard of evaluation of care and increasingly provide the basis for recommendations to clinicians, to providers of care and to policy makers. Results from this study will present the first assessment of the scope, quality and accessibility of mental health trials in low and middle-income countries.

## Competing interests

Lelia Duley is a partner in the Practihc project. Clive Adams, Prathap Tharyan and Lelia Duley have all contributed to the main Practihc project of trials in low and middle-income countries.

## Authors' contributions

RJSS wrote the first draft and further revision with input from all authors. MJ selected relevant trials from the CENTRAL downloads. PT is responsible for the data verification from the Christian Medical College, Vellore, India. LD is responsible for the main PRACTIHC survey, helped select and collate reports and design data extraction sheets. CEA helped design and manage the search, helped select and collate reports and design data extraction sheet. All authors read and approved the final manuscript.

## Pre-publication history

The pre-publication history for this paper can be accessed here:



## Supplementary Material

Additional File 1 PRACTIHC search. Other databases searched for the PRACTIHC project with dates and yield for all types of trials. Click here for file

Additional File 2 Mental Health Search Terms. The search terms used to search the original PRACTIHC sample for citations potentially relevant to mental health. Click here for file

Additional File 4 Extra Mental Health Data. The data collection form for the extra mental health terms. Click here for file

Additional File 3 Trials in low and middle-income countries. The main PRACTIHC data collection form. Click here for file

Additional File 5 High-Income Country Schizophrenia Data. The data collection form for high-income country schizophrenia trials. Click here for file

Additional File 6 Dummy Tables. This contains the dummy tables and figures, which will accommodate the final analysis. Click here for file

Additional File 7 Regional reporting categories for the Disease Control Priorities Project (DCPP). This shows how low and middle-income countries are divided up in terms of regions. Click here for file
