# Supplementary material for: Randomised trials relevant to mental health conducted in low and middle-income countries: protocol for a survey of studies published in 1991, 1995 and 2000 and assessment of their relevance
Source: BMC Psychiatry. 2006 Sep 26;6:40. doi: 10.1186/1471-244X-6-40 (PMC1609111; doi:10.1186/1471-244X-6-40)
Supplement: Additional File 1 — PRACTIHC search. Other databases searched for the PRACTIHC project with dates and yield for all types of trials. [file 1471-244X-6-40-S1.doc]

**PRACTIHC Search**

| **Region** | **Database** | **URL** |
| --- | --- | --- |
| **Global** | Cochrane’s CENTRAL database | http://www.mrw.interscience.wiley.com/cochrane |
| **Africa** | African Index Medicus | http://indexmedicus.afro.who.int/ |
|  | African Trials Register | <http://www.mrc.ac.za/ATR/> |
|  | Pascal (includes Francophone Africa) | [www.datastarweb.com](../www.datastarweb.com). |
|  | Scientific and Technical Egyptian Bibliographic Database (STEB) | <http://www.sti.sci.eg/enstinetdatabases.htm> |
| **Arabia** | ArabPsyNet | <http://www.arabpsynet.com/INDEX.ASP> |
|  | SaudMed | <http://www.smj.org.sa/smjmedbase.asp> |
| **- and Eastern Med Region** | Eastern Mediterranean Literature (WHO) | [http://www.emro.who.int/](http://www.emro.who.int/cgi-bin/wxis.exe/iah/?IsisScript=iah/iah.xic&base=imemr&lang=i) |
| **Asia - Central** | Parsmedline (Iran) | <http://www.parsmedline.net/> |
| **- Indian subcontinent** | IndMED (India) | http://indmed.nic.in/ |
| **- South-East** | Index Medicus for South-East Asia Region (IMSEAR) | <http://www.hellis.org/> |
|  | HERDIN (Philippines) | http://www.herdin.ph |
|  | KoreaMED | <http://www.koreamed.org/SearchBasic.php> |
|  | MEDLIP (Pakistan) | http://www.cpsp.edu.pk/Onlinedirectory/Medlip/ |
|  | Thai – AIDS/HIV | http://www.aidsthaidata.org/ |
|  | Thai – Index Medicus | <http://161.200.96.233/thaiim.html> |
|  | Thai – Thesis | <http://thesis.tiac.or.th/> |
| **China** | CAJ | http://online.eastview.com/index.jsp |
| **Europe** | Polska Bibliografia Lekarsko (Poland) | CD-Rom only |
|  | BiblioMedica (Czech Republic) | <http://www.aipberoun.cz/produkty_bm_en.htm> |
|  | Magyar Orvosi Bibliográfia (Hungary) | http://sunny.eski.hu:8080/cgi-bin/w1.sh |
|  | MediaSphere (Russia) | <http://www.mediasphera.aha.ru/english.htm>  <http://www.rmj.ru/main.htm>  <http://www.rusmedserv.com/> |
|  | Turkish Academic Network and Information Center (Turkey) | http://www.ulakbim.gov.tr/eng/ |
|  | Çukurova Psikiyatri Dizini (Turkey) | http://www.psikiyatridizini.org/ |
|  | Panteleimon (Ukraine) | <http://www.panteleimon.org/> |
| **Latin America** | LILACS (Latin America) | <http://www.bireme.br/iah2/homepagei.htm> |
