# Supplementary material for: Randomised trials relevant to mental health conducted in low and middle-income countries: protocol for a survey of studies published in 1991, 1995 and 2000 and assessment of their relevance
Source: BMC Psychiatry. 2006 Sep 26;6:40. doi: 10.1186/1471-244X-6-40 (PMC1609111; doi:10.1186/1471-244X-6-40)
Supplement: Additional File 2 — Mental Health Search Terms. The search terms used to search the original PRACTIHC sample for citations potentially relevant to mental health. [file 1471-244X-6-40-S2.doc]

Mental Health Search Terms

*schiz*

*mental*

*depres*

*alcohol*

*substance abuse*

*substance misuse*

*substance depend*

*substance withdraw*

*marijuana*

*amphetamine*

*cocaine*

*heroin*

*tobacco*

*smok*

*psych*

*mania*

*bipolar*

*anxi*

*obses*

*OCD*

*eating dis*

*anorexia*

*bulimia*

*personality dis*

*ADHD*

*attention def*

*learning dis*

*compuls*

*demen*

*behav*

*dissocia*

*factitious*

*developmental*

*neurotic*

*paranoi*

*somatoform*

*comport*

*esquiz*

*dépres*

*psic*

*psiq*

*folie*

*manía*

*ansie*

*démen*

**Logical –**or
