# Supplementary material for: Randomised trials relevant to mental health conducted in low and middle-income countries: protocol for a survey of studies published in 1991, 1995 and 2000 and assessment of their relevance
Source: BMC Psychiatry. 2006 Sep 26;6:40. doi: 10.1186/1471-244X-6-40 (PMC1609111; doi:10.1186/1471-244X-6-40)
Supplement: Additional File 4 — Extra Mental Health Data. The data collection form for the extra mental health terms. [file 1471-244X-6-40-S4.doc]

**Extra Mental Health Data**

| *Year* |  | *Citation ID* |  |
| --- | --- | --- | --- |

| **Age of participants** *****  *( WHO classification) list all that apply)* | 1. <4 yr 2. 5 - 14 yrs 3. 15- 29 yrs 4. 30-69 yrs | | 1. elderly people (>70yrs) 2. other 3. not stated |  |
| --- | --- | --- | --- | --- |
| **Quality grading *** | 1= Adequate concealment of allocation  2= Uncertain concealment of allocation | | 3= Clearly inadequate concealment of allocation |  |
| **If prevention trial**  1 =primary prevention, or 2= relapse prevention | |  | |  |
