# Supplementary material for: Randomised trials relevant to mental health conducted in low and middle-income countries: protocol for a survey of studies published in 1991, 1995 and 2000 and assessment of their relevance
Source: BMC Psychiatry. 2006 Sep 26;6:40. doi: 10.1186/1471-244X-6-40 (PMC1609111; doi:10.1186/1471-244X-6-40)
Supplement: Additional File 3 — Trials in low and middle-income countries. The main PRACTIHC data collection form. [file 1471-244X-6-40-S3.doc]

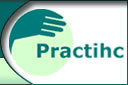
**Trials in low and middle-income countries**

| **Year** |  | **Citation ID** | | |  | **Data from abstract only**  **Data from title only** | |  | |
| --- | --- | --- | --- | --- | --- | --- | --- | --- | --- |
|  |  |  |  |  |  |  |  |  |  |
| **Type of report**   1. journal article, full report 2. journal letter | | 1. conference abstract in journal / supplement 2. conference proceedings 3. thesis / dissertation | | | | 1. book / book chapter 2. unpublished document 3. other …. ………..…………… | |  | |
| **Number of authors** (If collaborative group authorship, enter 99; if not stated enter 88) | | | | | | | |  | |
| **Country of authors ***  *(list each country from which there is an author)* | | First author | | | |  | | | |
|  |  | Corresponding *(if not 1st author)* | | | |  | | | |
|  |  | Other authors | | |  | | | | |
| **Language of report *** | | | | | |  | | | |
| **Age of participants** *****  *(list all that apply)* | | 1. babies/newborn (<1 yr) 2. young children (1 - 10 yrs) 3. older children (10 - 18 yrs) 4. pregnant/recently pregnant | | | | 1. adults (approx 18 - 60 yrs) 2. elderly people (>60 - 65 yrs) 3. other ………….……………… 4. not stated | | |  |
| **Sex of participants** | | 1. male only 2. female only | | | | 1. male + female 2. not stated | | |  |
| **Problem/s being addressed ***  *(list main features)* | |  | | | | | | | |
|  | | | 1 = prevention | | | 2 = treatment | |  | |
| **Number of participants randomised** | | | | | |  |  | | |
| **Unit of allocation** | | | 1. individual 2. cluster | | | 1. not clear or not stated | |  | |
| **If cluster design:** number of clusters | | | | | | | |  | |
| **Number of recruiting centres** (if not stated enter NS; if cluster trial enter NA) | | | | | | |  | | |
| **Name/s of recruiting countries**  (if not stated enter NS) | | | |  | | | | | |
| **Ethics committee approval** **mentioned** | | | | | | 0 = no  1 = yes | |  | |
| **Consent requested, or mentioned *** | | | | | | 0 = no  1 = yes | |  | |
| **Sequence generation**   1. computer randomisation 2. computer minimisation 3. random number tables | | | 1. coin toss, shuffling 2. quasi random - eg alternate allocation, days of week, hospital number | | | 1. other ………………………… 2. 'randomised' method not stated 3. controlled clinical trial 4. not an RCT *(exclude)* | |  | |
| **Allocation concealment**   1. telephone 2. prepared in pharmacy | | | 1. sealed numbered/coded envelopes/boxes 2. sealed envelopes/boxes 3. open, no concealment | | | 1. unclear from description 2. other ………..……..………… 3. not stated | |  | |

| **Setting of intervention** | | | 1. rural 2. urban | | 1. other ………………………… 2. not stated | | |  | |
| --- | --- | --- | --- | --- | --- | --- | --- | --- | --- |
| **Site of intervention**   1. hospital - ward or clinic | | | 1. primary care - eg GP, primary health centre 2. community - eg old folks home, own home | | 1. educational institution 2. other…………….………….… 3. not stated | | |  | |
| **Number of interventions arms** | | | | | | | |  | |
| **Type of arms** | | | 1. parallel arms 2. parallel, factorial | | 1. crossover 2. not stated | | |  | |
| **Blinding of intervention** | | | 1. single blind 2. double blind | | 1. other ………………..……...… 2. not stated 3. no blinding | | |  | |
| **Allocated interventions ***  *(list as in trial report, for all* *groups*) | | | |  | | | |  | |
| **Duration of intervention - same in all groups?** | | | | | 0 = no 2 = not stated  1 = yes | | |  | |
| **Duration of intervention*** (list for each group) | | | | | | **group 1** | |  | |
| 1. <24 hours 2. 24 hours - 7 days 3. 8 days - 4 weeks 4. >4 weeks - 8 weeks | | | 1. >8 weeks - 6 months   6 = >6 months - 1 year  7 = >1 year – 5 years  8 = >5 years  9 = not stated | | | **group 2** | |  | |
|  |  |  |  |  |  | **group 3** | |  | |
|  |  |  |  |  |  | **group 4** | |  | |
|  |  |  |  |  |  | **group 5** | |  | |
| **Primary or main outcome***  If not stated, enter 'NS' | | | |  | | | | | |
| **Power calculation, sample size estimate?** ***** | | | | | 0 = no  1 = yes | | |  | |
| **Outcomes reported ***  (only list outcomes for which data are reported) |  | | | | | | | | |
| **Follow up - duration**   1. to discharge from hospital 2. <24 hours | | | 1. 24 hours - 7 days 2. 8 days - 4 weeks 3. >4 weeks - 8 weeks 4. >8 weeks - 6 months | | 1. >6 months - 1 year 2. >1 year - 5 years 3. >5 years 4. not stated | | |  | |
| **Number of participants with outcome data *** If not stated, enter 'NS' | | | | | | |  | | |
| **If cluster trial:** number of clusters with outcome data | | | | | | |  | |  |
| **Funding source/s ***  (list all that apply) | | 1. university 2. industry 3. government 4. research council | | | 1. other………………………….   ………………………………………  ………………………………………  6 = not stated | | |  | |
| **Countries of funding source**  (list all that apply) * | | | |  | | | | | |
