# Supplementary material for: Randomised trials relevant to mental health conducted in low and middle-income countries: protocol for a survey of studies published in 1991, 1995 and 2000 and assessment of their relevance
Source: BMC Psychiatry. 2006 Sep 26;6:40. doi: 10.1186/1471-244X-6-40 (PMC1609111; doi:10.1186/1471-244X-6-40)
Supplement: Additional File 5 — High-Income Country Schizophrenia Data. The data collection form for high-income country schizophrenia trials. [file 1471-244X-6-40-S5.doc]

**High-Income Country Schizophrenia Data**

| *Year* | |  | | *Citation ID* | | |  | *Data from abstract only?* | |  | | | |
| --- | --- | --- | --- | --- | --- | --- | --- | --- | --- | --- | --- | --- | --- |
| **Main Aims** | 1= treatment of primary disorder 3= relapse prevention  2= prevention 4= other | | | | | | | | | | |  |  |
| **Number randomised** |  | | | | | | | | | | | | |
| **Allocated interventions***(list as in trial report, for all* *groups*) | | | | | |  | | | |  | | | |
| Add name of drug | | | | | |  | | | |  | | | |
| **Number of participants with outcome data *** If not stated, enter 'NS' | | | | | | | | |  | | | | |
| **If cluster trial, number of clusters with outcome data** | | | | | | | | |  | |  | | |
| **Ethics committee approval mentioned** | | | | | | | | | **0 = no**  **1 = yes** | |  | | |
| **Consent requested, or mentioned *** | | | | | | | | | **0 = no**  **1 = yes** | |  | | |
| **Sequence generation**   1. computer randomisation 2. computer minimisation 3. random number tables | | | | | 1. coin toss, shuffling 2. quasi random - eg alternate allocation, days of week, hospital number | | | 1. other ………………………… 2. 'randomised' method not stated 3. controlled clinical trial 4. not an RCT *(exclude)* | |  | | | |
| **Allocation concealment**   1. telephone 2. prepared in pharmacy | | | | | 1. sealed numbered/coded envelopes/boxes 2. sealed envelopes/boxes 3. open, no concealment | | | 1. unclear from description 2. other ………..……..………… 3. not stated | |  | | | |
| **Quality grading *** | | | | | 1= Adequate concealment of allocation  2= Uncertain concealment of allocation | | | 3= Clearly inadequate concealment of allocation | |  | | | |
| **Blinding of intervention** | | | | | 1. single blind 2. double blind | | | 1. other ………………..……...… 2. not stated 3. no blinding | |  | | | |
| **Outcomes reported ***  (only list outcomes for which data are reported) | | |  | | | | | | | | | | |
