# Supplementary material for: Randomised trials relevant to mental health conducted in low and middle-income countries: protocol for a survey of studies published in 1991, 1995 and 2000 and assessment of their relevance
Source: BMC Psychiatry. 2006 Sep 26;6:40. doi: 10.1186/1471-244X-6-40 (PMC1609111; doi:10.1186/1471-244X-6-40)
Supplement: Additional File 6 — Dummy Tables. This contains the dummy tables and figures, which will accommodate the final analysis. [file 1471-244X-6-40-S6.doc]

**Dummy Tables**

**Contents -**

A. Description

B. Trends and associations

C. Comparison with schizophrenia studies from high income countries

D. Comparison with burden of disease estimates

**Key -**

*Economic groups*

HIC High Income Countries

UMIC Upper Middle Income Countries

LMIC Lower Middle Income Countries

LIC Low Income Countries

L/MIC All Lower and Middle Income Countries

*Broad geographical regions*

EAP East Asia and Pacific

ECA Europe and Central Asia

LAC Latin America and Caribbean

MENA Middle East and North Africa

ASS Sub-Saharan Africa

SA South Asia

O other

**A. Description**

**Table 1:** Type of report

| **Type of report** | **Reports**  **N (%)** |
| --- | --- |
| Journal article, full report |  |
| Journal letter |  |
| Conference abstract in journal/supplement |  |
| Conference proceedings |  |
| Thesis/dissertation |  |
| Book/book chapter |  |
| Unpublished document |  |
| Other |  |

**Table 2:** Country of first author

| **Country of first author** | **Reports**  **N (%)** |
| --- | --- |
| Low income |  |
| Lower middle income |  |
| Upper middle income |  |
| High income |  |
| EAP |  |
| ECA |  |
| LAC |  |
| MENA |  |
| ASS |  |
| SA |  |
| Other |  |
| Unknown |  |

**Table 3:** Country of recruitment

| **Country of first recruitment** | **Trials**  **N (%)** |
| --- | --- |
| Low income |  |
| Lower middle income |  |
| Upper middle income |  |
| High income |  |
| EAP |  |
| ECA |  |
| LAC |  |
| MENA |  |
| ASS |  |
| SA |  |
| Other |  |
| Unknown |  |

**Table 4**: Language of full report

| **Language of full report** | **Reports**  **N (%)** |
| --- | --- |
| French |  |
| Malay |  |
| Portuguese |  |
| Bengali |  |
| Arabic |  |
| Russian |  |
| Spanish |  |
| Hindustani |  |
| English |  |
| Mandarin |  |
| TOTALS |  |

**Table 5:** Age of participants

| **Age of participants** | **Trials**  **N (%)** |
| --- | --- |
| ≤4 |  |
| 5-14 |  |
| 15-29 |  |
| 30-69 |  |
| ≥70 |  |
| other |  |
| Not stated |  |

**Table 6:** Sex of participants

| **Sex of participants** | **Trials**  **N (%)** |
| --- | --- |
| Male only |  |
| Female only |  |
| Male and female |  |
| Not stated |  |

**Table 7:** Problems being addressed

| **Problems being addressed** | **Trials**  **N (%)** |
| --- | --- |
| Unipolar depression |  |
| Bipolar depression |  |
| schizophrenia |  |
| Alcohol use disorders |  |
| Alzheimer’s and other dementias |  |
| Drug use disorders |  |
| Post Traumatic Stress Disorder |  |
| Obsessive Compulsive Disorder |  |
| Panic disorder |  |
| Other |  |

**Table 8:** Main Aims

| **Main Aims** | **Trials**  **N (%)** |
| --- | --- |
| Treatment |  |
| Prevention |  |
| Relapse prevention |  |
| Other |  |
| Not stated |  |

**Table 9:** Number randomised

| **Number randomised** | **Trials**  **N (%)** |
| --- | --- |
| <50 |  |
| 50-99 |  |
| 100-499 |  |
| 500-999 |  |
| ≥1000 |  |

**Table 10:** Type of intervention

| **Type of intervention** | **Trials**  **N (%)** |
| --- | --- |
| Pharmacological  Drug  Non drug |  |
| Psychological/ social |  |

**Table 11:** Setting

| **Setting** | **Trials**  **N (%)** |
| --- | --- |
| rural |  |
| urban |  |
| other |  |
| Not stated |  |

**Table 12:** Site

| **Site** | **Trials**  **N (%)** |
| --- | --- |
| Hospital |  |
| Primary care |  |
| Educational institution |  |
| Other |  |

**Table 13:** Duration of Intervention

| **Duration of intervention** | **Trials**  **N (%)** |
| --- | --- |
| <24 hrs |  |
| 24hrs- 7days |  |
| 8 days -4 weeks |  |
| 4 weeks- 8 weeks |  |
| > 8 weeks |  |
| 6 months- 1year |  |
| 1- 5 years |  |
| > 5 years |  |
| Not stated |  |

**Table 14:** Follow up duration

| **Follow up duration** | **Trials**  **N (%)** |
| --- | --- |
| To discharge from hospital |  |
| <24 hrs |  |
| 24hrs- 7days |  |
| 8 days -4 weeks |  |
| 4 weeks- 8 weeks |  |
| 8 weeks- 6months |  |
| 6 months- 1year |  |
| 1- 5 years |  |
| > 5 years |  |
| Not stated |  |

**Table 15:** Funding Source

| **Funding source** | **Trials**  **N (%)** |
| --- | --- |
| university |  |
| industry |  |
| government |  |
| Research council |  |
| other |  |
| Not stated |  |

**Table 16:** Country of funding.

| **Country of funding** | **Trials**  **N (%)** |
| --- | --- |
| Low income |  |
| Lower middle income |  |
| Upper middle income |  |
| High income |  |
| Not stated |  |

**Table 17:** Ethics approval mentioned in report

| **Ethics committee approval mentioned** | **Trials**  **N (%)** |
| --- | --- |
| Yes |  |
| No, based on full report |  |

**Table 18:** Consent mentioned in report

| **Consent requested or mentioned** | **Trials**  **N (%)** |
| --- | --- |
| Yes |  |
| No, based on full report |  |

**Table 19:** Types of outcome reported

| **Outcome** | **Trials**  **N (%)** |
| --- | --- |
| global impression |  |
| mental state |  |
| behaviour |  |
| service outcomes |  |
| quality of life |  |
| adverse effects |  |
| economic outcomes |  |
| Social functioning |  |
| cognitive |  |
| physical tests |  |
| psychological tests |  |
| compliance/ attitudes to treatment |  |
| development and learning |  |
| withdrawal and craving/ other |  |
| Total trials |  |

**Table 20:** Concealment of allocation score

| **Quality Score** | **Trials**  **N (%)** |
| --- | --- |
| Adequate |  |
| Unclear |  |
| Clearly inadequate |  |

**Table 21: Sequence generation**

| **Sequence Generation** | **Trials**  **N (%)** |
| --- | --- |
| Randomised (as in items 1-4 of data extraction sheet) |  |
| Quasi randomised |  |
| Other |  |
| Method not mentioned |  |
| Controlled Clinical Trial |  |

**Table 22: Blinding**

| **Blinding** | **Trials**  **N (%)** |
| --- | --- |
| Single blind |  |
| Double blind |  |
| Other |  |
| Not stated |  |

**Table 23:** Proportion with outcome data

| **Number randomised** | **Trials**  **N (%)** |
| --- | --- |
| <50% |  |
| 50-60% |  |
| 60-70% |  |
| 70-80% |  |
| 80-90 |  |
| 90-95% |  |
| >95% |  |

**Table 24:** Accessibility of full report

| **Accessible on Medline** | **Trials**  **N (%)** |
| --- | --- |
| **Yes** |  |
| **No** |  |

1. **Trends and associations**

**Figure 1:** Number of citations by year

No of citations

Year of publication

**Figure 2:** Number of participants randomized by year

No randomised

Year of publication

**Figure 3:** Number of citations by year and economic group

……. low income

------- lower middle income

____ upper middle income

No of citations

Year of publication

**Figure 4:** Number randomized by year and economic group

……. low income

------- lower middle income

____ upper middle income

No randomised

Year of publication

**Figure 5:** Number of citations by year and geographic region

No of citations

Year of publication

**Figure 6:** Number randomized by year and geographic region

No randomised

Year of publication

**Figure 7:** Grading of concealment of allocation by year

……. Indexed on Medline

------- Not Indexed on Medline

Number of studies

Year of publication

**Figure8:** Type of intervention by year

……. biological

------- psycho/social

Number of studies

Year of publication

**Figure 9:** Accessibility by Year

……. Indexed on Medline

------- Not indexed on Medline

Number of studies

Year of publication

**Table 25:** Concealment of allocation by economic groups and geographic regions.

|  | **adequate concealment**  **N** |
| --- | --- |
| Economic status of country of participants |  |
| Low income |  |
| Lower middle income |  |
| Upper middle income |  |
| High income |  |
| Mixed high and low/middle income |  |
|  |  |
| Geographic region of country of participants |  |
| EAP |  |
| ECA |  |
| LAC |  |
| MENA |  |
| ASS |  |
| SA |  |
| Other |  |
| Unknown |  |

**Table 26:** Accessibility by economic groups and geographic regions

| **Country** | **Indexed** | **Not Indexed** |
| --- | --- | --- |
| Low income |  |  |
| Lower middle income |  |  |
| Upper middle income |  |  |
| High income |  |  |
| EAP |  |  |
| ECA |  |  |
| LAC |  |  |
| MENA |  |  |
| ASS |  |  |
| SA |  |  |
| Other |  |  |
| Unknown |  |  |

**Table 27:** Number randomized by economic groups and geographic regions

| **Country** | **Number randomised**  **N (%)** |
| --- | --- |
| Low income |  |
| Lower middle income |  |
| Upper middle income |  |
| High income |  |
| EAP |  |
| ECA |  |
| LAC |  |
| MENA |  |
| ASS |  |
| SA |  |
| Other |  |
| Unknown |  |

**Table 28:** Association of accessibility with language and quality score

| **Accessible by Medline** | **English**  **N** | **Non English**  **N** | **Average Quality score**  **N** |
| --- | --- | --- | --- |
| Yes |  |  |  |
| No |  |  |  |
| TOTALS |  |  |  |

**Table 29: Association of quality with accessibility**

| **Quality** | **Accessible on Medline** | **Not Accessible on Medline** |
| --- | --- | --- |
| Average Quality Score |  |  |
| TOTALS | XXX (100) |  |

**Table 30:** Association of source of funding with quality and language

| **Sources** | **English**  **N** | **Non English**  **N** | **Concealment of allocation score**  **N** |
| --- | --- | --- | --- |
| University |  |  |  |
| Industry |  |  |  |
| Governement |  |  |  |
| Research council |  |  |  |
| Other |  |  |  |
| Not stated |  |  |  |
| TOTALS |  |  |  |

**Part C: Comparison with HIC Schizophrenia Trials including trends over time**

**Table 31: Concealment of allocation grading**

| **Blinding** | **H I C**  **N (%)** | **All LMIC**  **N (%,)** |
| --- | --- | --- |
| Adequate |  |  |
| Unsure |  |  |
| Clearly inadequate |  |  |

**Table 32: Number randomised**

| Number randomised | **H I C**  N | **All LMIC**  N |
| --- | --- | --- |
| <50 |  |  |
| 50-99 |  |  |
| 100-499 |  |  |
| 500-999 |  |  |
| ≥1000 |  |  |

**Table 33: Type of intervention**

| **Type of intervention** | **H I C**  **N (%)** | **All LMIC**  **N (%)** |
| --- | --- | --- |
| **Pharmacological**  Typical  Atypical  Other  Not clear |  |  |
| **Psychological** |  |  |
| **Social** |  |  |

**Table 34: Accessibility**

| Indexed in Medline | **H I C**  **N (%)** | **All LMIC**  **N (%)** |
| --- | --- | --- |
| Yes |  |  |
| No |  |  |
| **TOTALS** | **XXX (100)** |  |

**Part D: Comparison with Estimated Need.**

**Table 35:** Research: need ratio for cause, age group and gender group by economic groups

| **Research: Need**  **No randomized/YLD** | **L/MIC** | MI | LIC | EAP | ECA | LAC | MENA | ASS | SA | Other |
| --- | --- | --- | --- | --- | --- | --- | --- | --- | --- | --- |
| Unipolar depression |  |  |  |  |  |  |  |  |  |  |
| Bipolar depression |  |  |  |  |  |  |  |  |  |  |
| Schizophrenia |  |  |  |  |  |  |  |  |  |  |
| Alcohol use disorders |  |  |  |  |  |  |  |  |  |  |
| Alzheimer’s and other dementias |  |  |  |  |  |  |  |  |  |  |
| Drug use disorders |  |  |  |  |  |  |  |  |  |  |
| PTSD |  |  |  |  |  |  |  |  |  |  |
| OCD |  |  |  |  |  |  |  |  |  |  |
| Panic disorder |  |  |  |  |  |  |  |  |  |  |
| Other |  |  |  |  |  |  |  |  |  |  |
| Male only |  |  |  |  |  |  |  |  |  |  |
| Female only |  |  |  |  |  |  |  |  |  |  |
| Male and female |  |  |  |  |  |  |  |  |  |  |
| Not stated |  |  |  |  |  |  |  |  |  |  |
| <4 yr |  |  |  |  |  |  |  |  |  |  |
| 5-14 |  |  |  |  |  |  |  |  |  |  |
| 15-29 |  |  |  |  |  |  |  |  |  |  |
| 30-69 |  |  |  |  |  |  |  |  |  |  |
| elderly |  |  |  |  |  |  |  |  |  |  |
| other |  |  |  |  |  |  |  |  |  |  |
| not stated |  |  |  |  |  |  |  |  |  |  |
