# Supplementary material for: Randomised trials relevant to mental health conducted in low and middle-income countries: protocol for a survey of studies published in 1991, 1995 and 2000 and assessment of their relevance
Source: BMC Psychiatry. 2006 Sep 26;6:40. doi: 10.1186/1471-244X-6-40 (PMC1609111; doi:10.1186/1471-244X-6-40)
Supplement: Additional File 7 — Regional reporting categories for the Disease Control Priorities Project (DCPP). This shows how low and middle-income countries are divided up in terms of regions. [file 1471-244X-6-40-S7.doc]

Regional reporting categories for the Disease Control Priorities Project (DCPP).

| DCPP region | Countries included |
| --- | --- |
| East Asia and Pacific | American Samoa, Cambodia, China, DPR Korea, Fiji, Indonesia, Kiribati, Lao People's Democratic Republic, Malaysia, Marshall Islands, Micronesia (Federated States Of), Mongolia, Myanmar, Palau, Papua New Guinea, Philippines, Samoa, Solomon Islands, Thailand, Timor-Leste, Tonga, Vanuatu, Viet Nam |
| Europe and Central Asia | Albania, Armenia, Azerbaijan, Belarus, Bosnia And Herzegovina, Bulgaria, Croatia, Czech Republic, Estonia, Georgia, Hungary, Isle of Man, Kazakhstan, Kyrgyzstan, Latvia, Lithuania, Poland, Republic of Moldova, Romania, Russian Federation, Serbia and Montenegro, Slovakia, Tajikistan, The Former Yugoslav Republic Of Macedonia, Turkey, Turkmenistan, Ukraine, Uzbekistan |
| High Income | Andorra, Aruba, Australia, Austria, Bahamas, Bahrain, Belgium, Bermuda, Brunei Darussalam, Canada, Cayman Islands, Channel Islands, Cyprus, Denmark, Faeroe Islands, Finland, France, French Polynesia, Germany, Greece, Greenland, Guam, Iceland, Ireland, Israel, Italy, Japan, Kuwait, Liechtenstein, Luxembourg, Monaco, Netherlands, Netherlands Antilles, New Caledonia, New Zealand, Nothern Mariana Islands, Norway, Portugal, Qatar, Republic of Korea, San Marino, Singapore, Slovenia, Spain, Sweden, Switzerland, United Arab Emirates, United Kingdom, United States of America, United States Virgin Islands |
| Latin America and Caribbean | Antigua and Barbuda, Argentina, Barbados, Belize, Bolivia, Brazil, Chile, Colombia, Costa Rica, Cuba, Dominica, Dominican Republic, Ecuador, El Salvador, Grenada, Guatemala, Guyana, Haiti, Honduras, Jamaica, Mexico, Nicaragua, Panama, Paraguay, Peru, Puerto Rico, Saint Kitts And Nevis, Saint Lucia, Saint Vincent And The Grenadines, Suriname, Trinidad And Tobago, Uruguay, Venezuela |
| Middle East and North Africa | Algeria, Djibouti, Egypt, Iran (Islamic Republic Of), Iraq, Jordan, Lebanon, Libyan Arab Jamahiriya, Malta, Morocco, Occupied Palestinian Territory, Oman, Saudi Arabia, Syrian Arab Republic, Tunisia, Yemen |
| South Asia | Afghanistan, Bangladesh, Bhutan, India, Maldives, Nepal, Pakistan, Sri Lanka |
| Sub-Saharan Africa | Angola, Benin, Botswana, Burkina Faso, Burundi, Cameroon, Cape Verde, Central African Republic, Chad, Comoros, Congo, Côte d’Ivoire, Democratic Republic of the Congo, Equatorial Guinea, Eritrea, Ethiopia, Gabon, Gambia, Ghana, Guinea, Guinea-Bissau, Kenya, Lesotho, Liberia, Madagascar, Malawi, Mali, Mauritania, Mauritius, Mozambique, Namibia, Niger, Nigeria, Rwanda, Sao Tome and Principe, Senegal, Seychelles, Sierra Leone, Somalia, South Africa, Sudan, Swaziland, Togo, Uganda, United Republic of Tanzania, Zambia, Zimbabwe |
| Other | Anguilla, British Virgin Islands, Cook Islands, Falkland Islands, French Guiana, Gibraltar, Guadeloupe, Holy See, Martinique, Montserrat, Nauru, Niue, Pitcairn, Réunion, Saint Helena, Saint Pierre et Miquelon, Tokelau, Turks and Caicos Islands, Tuvalu, Wallis and Futuna Islands, Western Sahara |
